# Differentiation of IL-17-Producing Invariant Natural Killer T Cells Requires Expression of the Transcription Factor c-Maf

**DOI:** 10.3389/fimmu.2017.01399

**Published:** 2017-10-27

**Authors:** Jhang-Sian Yu, Michito Hamada, Shigeo Ohtsuka, Keigyou Yoh, Satoru Takahashi, Shi-Chuen Miaw

**Affiliations:** ^1^Graduate Institute of Immunology, College of Medicine, National Taiwan University, Taipei, Taiwan; ^2^Faculty of Medicine, Department of Anatomy and Embryology, University of Tsukuba, Ibaraki, Japan; ^3^Department of Respiratory Medicine, Graduate School of Comprehensive Human Sciences, University of Tsukuba, Ibaraki, Japan

**Keywords:** invariant natural killer T cells, c-Maf, IL-17, cytokine regulation, airway neutrophilia

## Abstract

c-Maf belongs to the large Maf family of transcription factors and plays a key role in the regulation of cytokine production and differentiation of T_H_2, T_H_17, T_FH_, and Tr1 cells. Invariant natural killer T (iNKT) cells can rapidly produce large quantity of T_H_-related cytokines such as IFN-γ, IL-4, and IL-17A upon stimulation by glycolipid antigens, such as α-galactosylceramide (α-GalCer). However, the role of c-Maf in iNKT cells and iNKT cells-mediated diseases remains poorly understood. In this study, we demonstrate that α-GalCer-stimulated iNKT cells express c-Maf transcript and protein. By using c-Maf-deficient fetal liver cell-reconstituted mice, we further show that c-Maf-deficient iNKT cells produce less IL-17A than their wild-type counterparts after α-GalCer stimulation. While c-Maf deficiency does not affect the development and activation of iNKT cells, c-Maf is essential for the induction of IL-17-producing iNKT (iNKT17) cells by IL-6, TGF-β, and IL-1β, and the optimal expression of RORγt. Accordingly, c-Maf-deficient iNKT17 cells lose the ability to recruit neutrophils into the lungs. Taken together, c-Maf is a positive regulator for the expression of IL-17A and RORγt in iNKT17 cells. It is a potential therapeutic target in iNKT17 cell-mediated inflammatory disease.

## Introduction

Natural killer T (NKT) cells are a subset of lymphocytes that bridge innate and adaptive immunity (1). They are special lymphocytes with both NK and T cell lineage markers (2) and are important immune cells that contribute to the defense against infectious diseases, allergy, cancer development, and autoimmunity (3–5). NKT cells are heterogeneous and can be divided into several subtypes based on their CD1d dependence and TCR repertoire (6). The most well-studied NKT cells are the CD1d-dependent invariant NKT (iNKT) cells which have a limited repertoire of αβTCR chains, allowing them to recognize glycolipid antigens [e.g., α-galactosylceramide (α-GalCer)] presented by CD1d molecules expressed by antigen-presenting cells (APCs) (7). Upon activation by α-GalCer, iNKT cells rapidly produce a large amount of cytokines, including IFN-γ (T_H_1 cytokine) and IL-4 and IL-13 (T_H_2 cytokine). In addition, recent studies have demonstrated that iNKT cells can also secret T_H_17-related cytokines, such as IL-17A, IL-17F, IL-21, and IL-22 (8–10). Furthermore, noncommitted iNKT cells can be induced to produce IL-17A when activated in the presence of IL-6, TGF-β, and IL-1β, thereby triggering airway neutrophilic inflammation (11).

c-Maf belongs to the large Maf family of transcription factors, which contain a basic leucine zipper domain. c-Maf is expressed at a high level in T_H_2 cells and is critical for inducing IL-4 expression and T_H_2 commitment (12, 13). Recent studies have demonstrated that c-Maf is also expressed in other immune cells, including T_H_17, T_FH_, and LPS-activated macrophages, and regulates the expression of IL-22, IL-21, and IL-10 (14–17). Previous study also demonstrated the capacity of c-Maf to bind to both *Il17a* and *Il17f* promoters in T_H_17 cells (18). Furthermore, c-Maf interacted with Sox5 and cooperatively induced T_H_17 cell differentiation *via* induction of RORγt (19). These finding strongly suggest that c-Maf plays a direct role in IL-17A production in T_H_17 cells. Recently, it is reported that c-Maf is one of the downstream target genes of the transcription factor PLZF in iNKT cells, as c-Maf expression is compromised in PLZF-deficient iNKT and γδNKT cells. Moreover, ectopic expression of c-Maf is sufficient to rescue IL-4 and IL-10 production by PLZF-deficient iNKT cells (20). However, the role of c-Maf in iNKT cells is not well known.

In this study, we used a genetic approach to demonstrate that c-Maf is expressed in α-GalCer-activated iNKT cells. We further show that c-Maf is required for the optimal expression of RORγt and IL-17, but not IFN-γ or IL-4, in iNKT cells and is essential for their ability to induce airway neutrophilic inflammation *in vivo*.

## Materials and Methods

### Mice and Cells

The 6- to 8-week-old female C57BL/6J mice from the Laboratory Animal Center of National Taiwan University College of Medicine were used as the source of naive CD4^+^ T cells and iNKT cells. All animals were housed under specific pathogen-free conditions. This study was carried out in strict accordance with the recommendations in the Guide for the Care and Use of Laboratory Animals of the National Institutes of Health (NIH). The protocol was approved by the Institutional Animal Care and Use Committee of National Taiwan University College of Medicine (Permit Number: 20140426). Water and food were provided sufficiently daily. All mice were sacrificed by CO_2_. Primary CD4^+^ T cells and iNKT cells cultured in complete RPMI 1640 medium (containing 10% FBS, 1 mM sodium pyruvate, 2 mM l-glutamine, 100 µM MEM non-essential amino acids, 10 mM HEPES, 5.5 × 10^−5^ M β-mercaptoethanol, 100 U/ml penicillin, and 100 µg/ml streptomycin).

### Generation of c-Maf-Deficient Reconstituted Mice

To generate the c-Maf-deficient reconstituted mice, the donor cells for hematopoietic reconstitution were collected from fetal livers of c-Maf-deficient fetuses (C57BL/6J-CD45.1) on embryonic day 14.5 (E14.5) and were transplanted into C57BL/6J-CD45.2 recipient mice, which were previously exposed to a single dose of X-ray irradiation (7 Gy) by tail vein injection. After 2 months for reconstitution of hematopoietic system, more than 90% hematopoietic cells were CD45.1^+^ confirmed by flow cytometry.

### Flow Cytometry and Cell Sorting

Mouse CD1d tetramers coupled to APC and loaded with PBS-57 (an iNKT cell ligand analog to α-GalCer) were provided by the NIH Tetramer Core Facility. Fluorochrome-labeled monoclonal antibodies (mAbs) specific to mouse CD45.1 (A20), CD45.2 (104), TCRβ (H57-597), CD4 (RM4-5), NK1.1 (PK136), Gr-1 (RB6-8C5), CD11b (M1/70), CD69 (H1.2F3), IFN-γ (XMG1.2), IL-4 (11B11), and IL-17A (TC11-18H10.1) were all purchased from BioLegend. c-Maf mAb (sym0F1) was purchased from eBioscience. For intracellular cytokine staining, cells were incubated with PMA (50 ng/ml), ionomycin (500 ng/ml), and brefeldin A (10 µg/ml; Sigma) for 3 h. After preincubation with unconjugated anti-CD16/32 (2.4G2; BioLegend) mAb, cells were stained for surface antigen and then fixed and permeabilized using the IC fixation buffer and permeabilization buffer (eBioscience) followed by incubation with anti-cytokine or isotype control Abs. FACS data were acquired by BD LSRFortessa (BD Biosciences) and analyzed with FlowJo software. For cell sorting, murine naive CD4^+^CD62L^hi^ T cells and CD1d/PBS-57^+^TCRβ^+^ iNKT cells were sorted by FACSAria IIIu (BD Biosciences).

### *In Vitro* T_H_ Cell Differentiation

Naive CD4^+^ T cells (CD4^+^CD62L^hi^) were prepared from spleens and peripheral lymph nodes (LNs) of 5- to 6-week-old wild-type (WT) female C57BL/6J mice, and stained with PE-CD4 (RM4-5) and FITC-CD62L (MEL-14) Abs (eBioscience), and purified by cell sorter (FACS Aria IIIu; BD Biosciences). Naive CD4^+^ T cells were seeded at 1 × 10^6^ cells/ml in complete RPMI 1640 medium and stimulated with plate-bound anti-CD3 (1 µg/ml; 2C11) mAb under T_H_0, T_H_1 (1 ng/ml of IL-12 and 10 µg/ml of anti-IL-4 mAb), T_H_2 (10 ng/ml of IL-4 and 10 µg/ml of anti-IFN-γ mAb), or T_H_17-skewing (20 ng/ml of IL-6, 2.5 ng/ml of TGF-β, 10 µg/ml of anti-IFN-γ, and 10 µg/ml of anti-IL-4) medium in the presence of γ-irradiated splenocytes (2,000 rad) as APCs for 5 days. Under T_H_0, T_H_1, and T_H_2-skewing conditions, human IL-2 (100 U/ml) was supplied with fresh medium daily; however, for T_H_17-skewing condition, human IL-2 (50 U/ml) was added on day 0 only. The skewed T_H_ cells were restimulated on anti-CD3-coated plate (1 µg/ml) for 24 h on day 5.

### *In Vivo* Stimulation of iNKT Cells

α-Galactosylceramide (2 µg in 100 µl PBS) or vehicle (DMSO in 100 µl PBS) was injected into the tail veins of WT or c-Maf knockout (KO) reconstituted mice. Splenocytes were collected 2 h after injection and cultured in complete RPMI 1640 medium (T cell culture medium; RPMI 1640 with 10% FBS, HEPES, penicillin and streptomycin, sodium pyruvate, nonessential amino acids, l-glutamine, and β-mercaptoethanol) in the presence of brefeldin A (10 µg/ml) for 2 h, and intracellular cytokine staining for IFN-γ, IL-4, IL-17A in iNKT cells was performed.

### *In Vitro* Induction of IL-17-Producing iNKT Cells

Splenocytes from WT or c-Maf KO reconstituted mice were pre-incubated on the anti-IgM/IgA/Ig light chain κ antibody-coated plates (10 µg/each Ab; BioLegend) to deplete B cells at room temperature for 45 min. After B cell depletion, WT or c-Maf KO splenocytes were stimulated with α-GalCer (100 ng/ml) to expand iNKT population for 5 days. CD45.1^+^ CD45.2^−^ CD1d/PBS-57^+^ TCRβ^+^ iNKT cells were purified by cell sorter (FACSAria IIIu; BD Biosciences). Purified WT or c-Maf KO iNKT cells were stimulated with plate-bound anti-CD3 (2 µg/ml) and anti-CD28 (5 µg/ml) in the presence of IL-6 (20 ng/ml), TGF-β (1 ng/ml), IL-1β (10 ng/ml; PeproTech), anti-IFN-γ (10 µg/ml), and anti-IL-4 (10 µg/ml) mAbs on 96-well plates (50,000 cells/well) for 4 days to induce IL-17-producing iNKT cells.

### Quantitative Real-time PCR

Total RNA was extracted with TRIzol Reagent (Invitrogen) according to the manufacturer’s instructions, and the cDNA was generated using SuperScript III Reverse Transcriptase (Invitrogen) or MMLV High Performance Reverse Transcriptase (epicentre). Standard SYBR Green real-time PCR was performed using and a IQ5 PCR detection system (Bio-Rad) or PikoReal Real-Time PCR system (Thermo Scientific). Relative expression of the target gene was normalized to β-actin and calculated as 2^−(Ct^ *^target ^*^− Ct^ *^β-actin^*^)^. The Ct represents the threshold cycle for each target or reference gene determined by Bio-Rad iQ5 or Thermo PikoReal Software 2.2. The relative target gene expression was calculated by using the 2^−ΔΔCt^ method.

### *In Vivo* Airway Neutrophilia Experiments

Wild-type C57BL/6J (6- to 8-week old) mice were anesthetized with Escain^®^ (Isoflurane) and then injected with 50,000 WT or c-Maf-deficient IL-17-producing iNKT cells *via* intratracheal route. Two days postinjection, the mice were sacrificed and the trachea cannulated, the airways were lavaged five times with 1 ml ice-old PBS and the bronchoalveolar lavage (BAL) collected.

### Statistical Analysis

Statistical analyses were performed with unpaired, two-tailed Student’s *t*-tests. A value of *P* < 0.05 was considered statistically significant.

## Results

### α-GalCer-Activated iNKT Cells Express c-Maf Transcript and Protein

**Figure 1:**
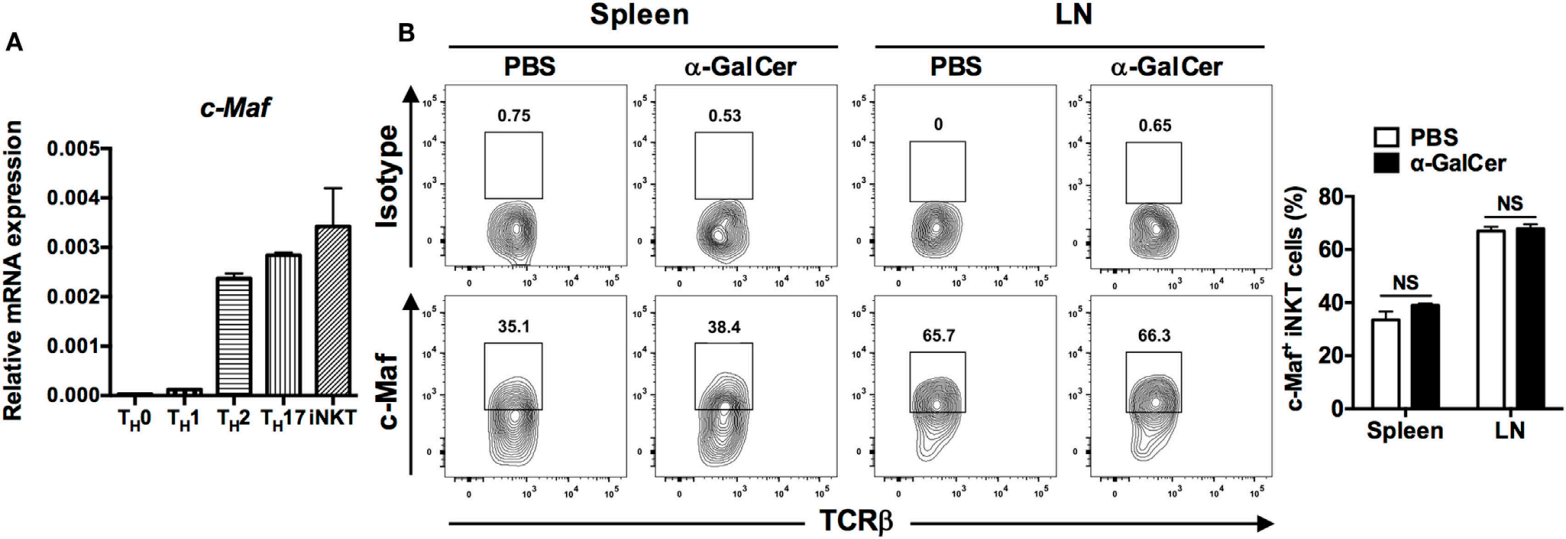
α-Galactosylceramide (α-GalCer)-stimulated invariant natural killer T (iNKT) cells express c-Maf. **(A)** T_H_0, T_H_1, T_H_2, and T_H_17 cells were re-stimulated with plate-bound anti-CD3 monoclonal antibody (mAb) for 24 h. Purified CD1d/PBS-57^+^TCRβ^+^ iNKT cells were cultured *in vitro* for 3 days. The transcript levels of c-Maf were determined by quantitative PCR and normalized against β-actin expression in each sample (*n* = 3). **(B)** α-GalCer or PBS was injected into the tail veins of WT C57BL/6J mice. Splenocytes and lymph node (LN) cells of the injected mice were collected 2 h later and subjected first to surface staining with PBS-57-loaded CD1d tetramer, anti-TCR-β mAb, and then intracellular staining for c-Maf. Representative FACS plots are shown. NS, not significant, determined by unpaired *t*-test, two-tailed (*n* = 3).

c-Maf is expressed in T_H_2, T_H_17, T_FH_, and LPS-activated macrophages (12–17). However, the expression of c-Maf in iNKT cells has yet to be characterized. To examine the expression of c-Maf in iNKT cells, CD1d/PBS-57^+^TCRβ^+^ cells were sorted from the splenocytes of WT C57BL/6J mice and cultured with complete RPMI 1640 medium for 3 days *in vitro*. Quantitative PCR results showed that iNKT cells expressed a level of c-Maf transcript that was much higher than that of anti-CD3-restimulated T_H_0 and T_H_1 cells, but comparable to that of anti-CD3-restimulated T_H_2 and T_H_17 cells (Figure 1A). To confirm the expression of c-Maf *in vivo*, WT mice were treated with α-GalCer and their splenocytes and LN cells were subjected first to surface staining with PBS-57-loaded CD1d tetramer and anti-TCRβ mAb, and then intracellular staining for c-Maf and indicated cytokines. There was a higher percentage of c-Maf-expressing iNKT cells in the peripheral LNs (67.0 ± 1.6%) than spleens (33.5 ± 3.2%) in vehicle (PBS)-treated mice. However, α-GalCer stimulation did not affect the percentages of c-Maf-expressing iNKT cells in either spleens (38.9 ± 0.7%) or LNs (67.9 ± 1.6%) (Figure 1B). Interestingly, c-Maf is expressed in IFN-γ, IL-4 or IL-17-producing iNKT cells (data not shown). Taken together, these results demonstrate that α-GalCer-activated iNKT cells express c-Maf mRNA and protein.

### c-Maf Positively Regulates IL-17A Production in α-GalCer-Activated iNKT Cells

**Figure 2:**
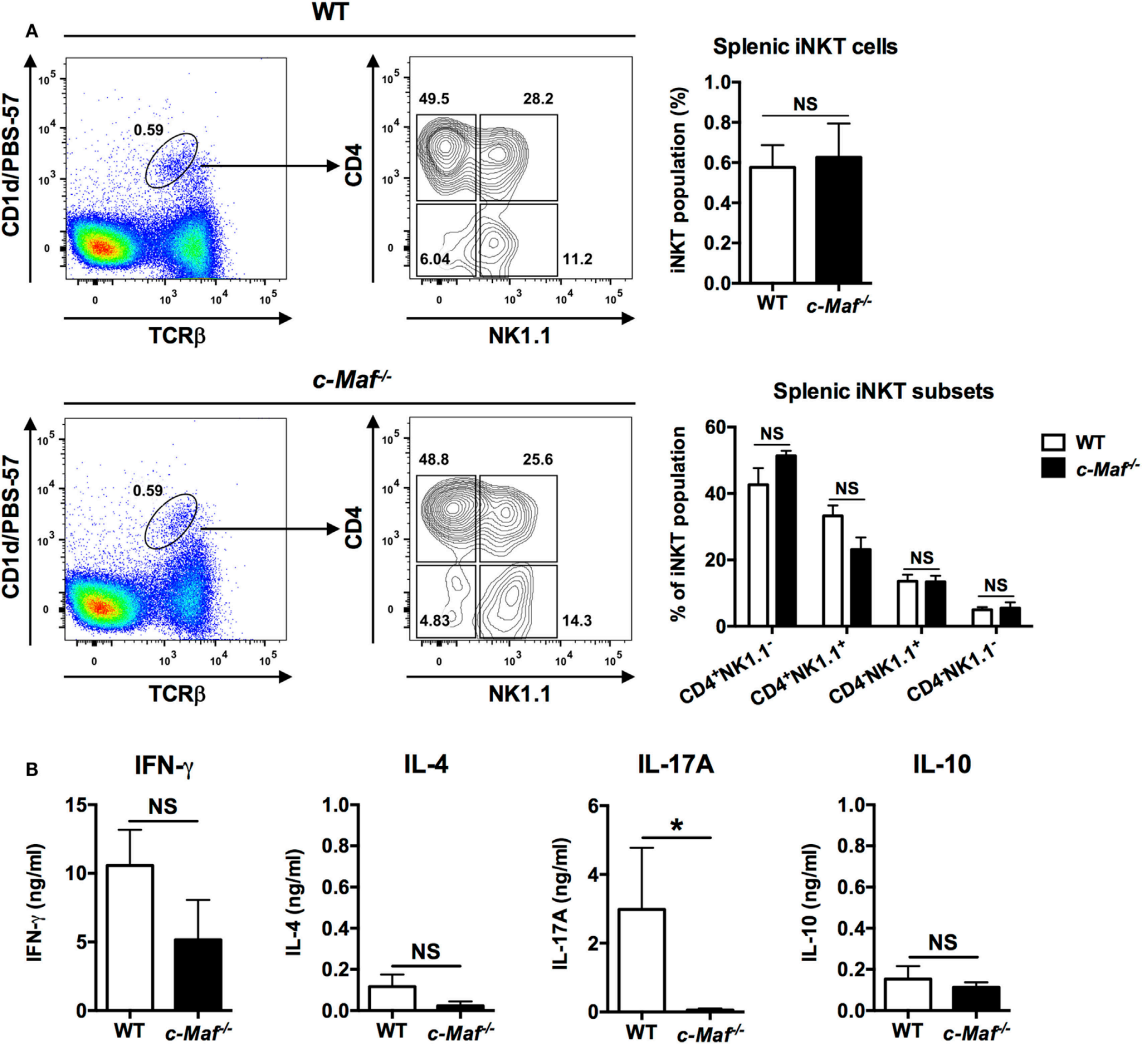
Invariant natural killer T (iNKT) cells from c-Maf knockout (KO)-reconstituted mice do not produce IL-17A upon α-galactosylceramide (α-GalCer) stimulation. **(A)** Total splenocytes were isolated from wild-type (WT) or c-Maf KO reconstituted mice and stained with PBS-57-loaded CD1d tetramer and monoclonal antibodies (mAbs) specific for TCR-β, CD4, and NK1.1. The percentages of iNKT cells among splenocytes and the percentage of the four subsets of iNKT cells (defined by NK1.1 and CD4) among total iNKT cells are determined by flow cytometry. Representative FACS plots are shown. NS, not significant, determined by unpaired *t*-test, two-tailed (*n* = 3). **(B)** The splenocytes were also stimulated with α-GalCer (100 ng/ml) for 4 days *in vitro*. The levels of IFN-γ, IL-4, IL-17A, and IL-10 in the culture supernatants were measured by ELISA. NS, not significant, *P < 0.05 determined by unpaired *t*-test, two-tailed (*n* = 3).

c-Maf KO mice are embryonic lethal due to anemia (21). We therefore reconstituted WT C57BL/6J mice with fetal liver cells from c-Maf KO embryos. The percentage of the iNKT population among splenocytes was comparable between WT and c-Maf KO reconstituted mice, indicating that c-Maf deficiency does not affect the development and homeostasis of iNKT cells in the spleen. Furthermore, the distribution of major iNKT subsets based on the expression of CD4 and NK1.1 was not affected by c-Maf deficiency (Figure 2A).

To investigate the role of c-Maf in iNKT cells, we stimulated WT and c-Maf KO splenocytes with α-GalCer *in vitro* and quantified their production of cytokines by ELISA. We found that splenocytes from reconstituted c-Maf KO mice produced significantly less IL-17A compared to WT splenocytes. By contrast, the levels of IFN-γ, IL-4, and IL-10 were comparable between WT and c-Maf KO splenocytes (Figure 2B). Since only iNKT cells respond to α-GalCer, our results indicate that c-Maf is a positive regulator for IL-17A production in α-GalCer-activated iNKT cells.

**Figure 3:**
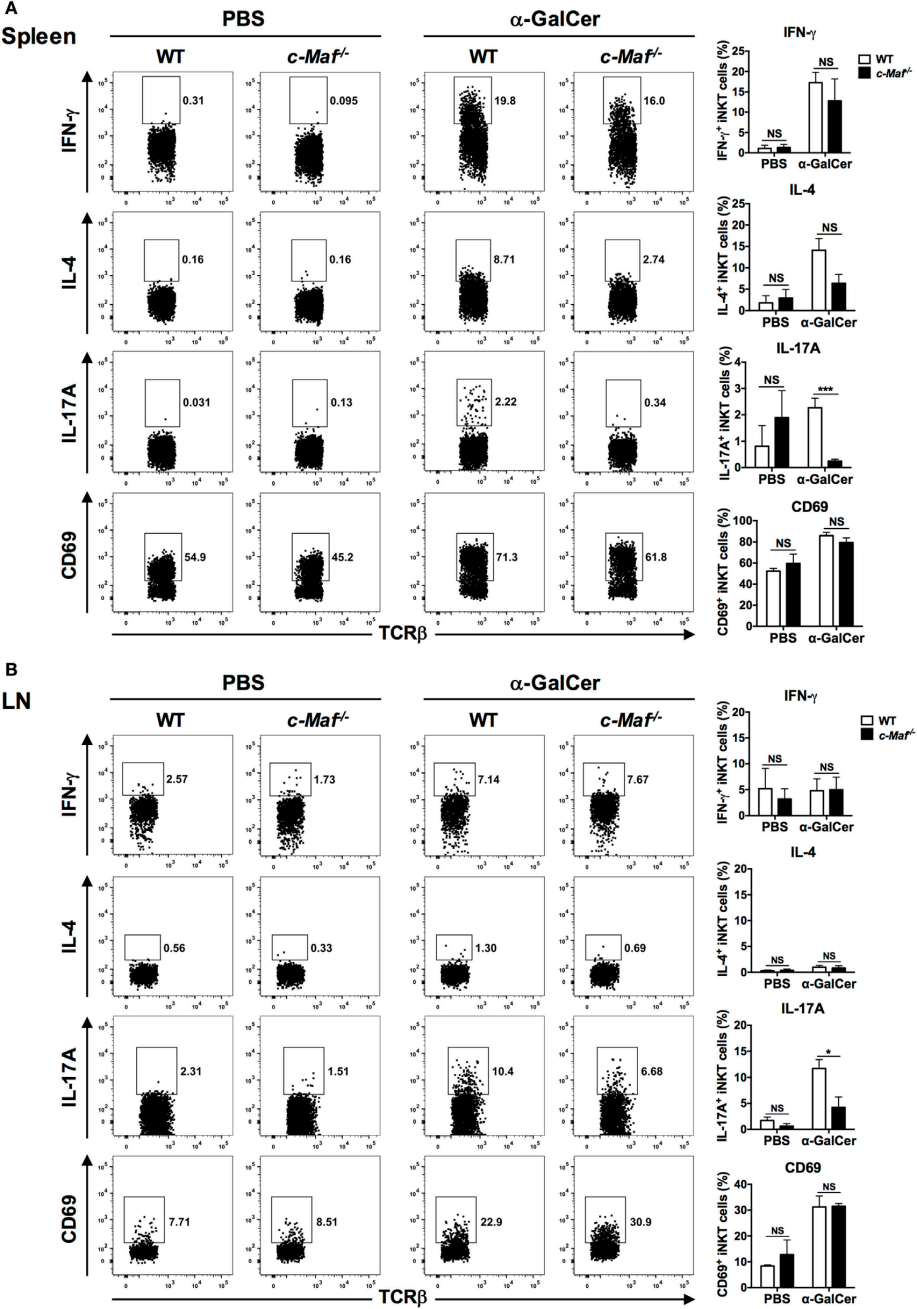
c-Maf deficiency impairs the production of IL-17A by invariant natural killer T (iNKT) from α-galactosylceramide (α-GalCer)-treated mice. α-GalCer or PBS was injected into the tail veins of wild-type (WT) or c-Maf knockout (KO) reconstituted mice. Splenocytes **(A)** and lymph node (LN) cells **(B)** of the injected mice were collected 2 h later and subjected first to surface staining with PBS-57-loaded CD1d tetramer, anti-TCR-β and CD69 monoclonal antibodies (mAbs), and then intracellular staining for indicated cytokines. Representative FACS plots are shown. NS, not significant, *P < 0.05; ***P < 0.001 determined by unpaired *t*-test, two-tailed (*n* = 3 in PBS-treated group; *n* = 6 in α-GalCer-treated group).

To address the role of c-Maf in regulating IL-17A production by iNKT cells *in vivo*, WT and c-Maf KO reconstituted mice were given α-GalCer intravenously. iNKT cells in the spleen and peripheral LNs were analyzed with intracellular cytokine staining. While c-Maf deficiency did not affect the activation state (CD69 expression) of splenic and LN iNKT cells, it markedly reduced their expression of IL-17A (Figure 3). These results together demonstrate that c-Maf positively regulates IL-17A production in α-GalCer-stimulated iNKT cells both *in vitro* and *in vivo*.

### c-Maf-Deficiency Deters the Polarization of iNKT17 Cells *In Vitro*

**Figure 4:**
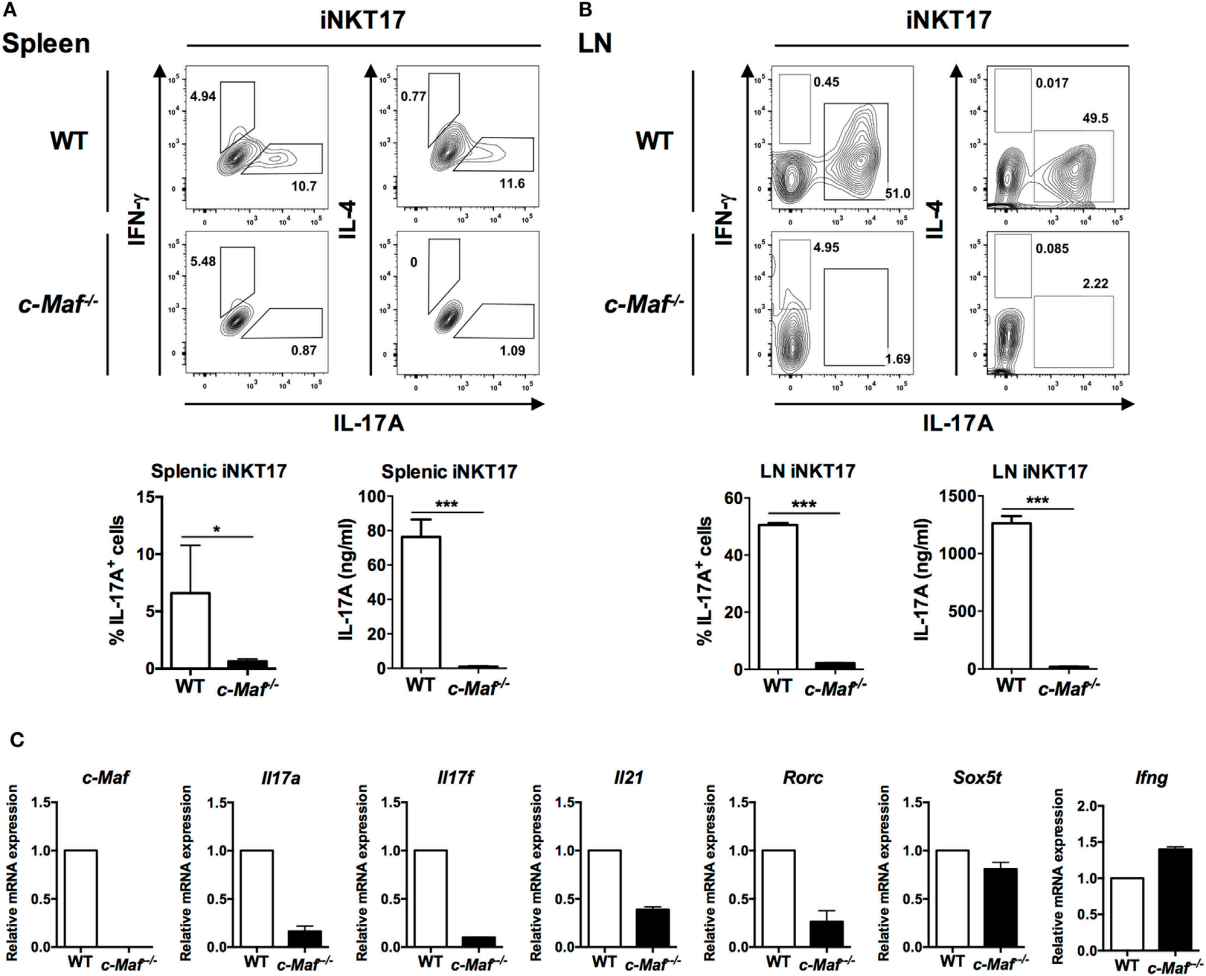
Splenic and lymph node (LN) c-Maf knockout (KO) invariant natural killer T (iNKT) 17 cells express less IL-17A than wild-type (WT) counterparts. Splenic **(A)** and LN **(B)** iNKT cells from WT or c-Maf KO reconstituted mice expanded *in vitro* with α-galactosylceramide (α-GalCer) for 5 days, sorted, and stimulated with anti-CD3/anti-CD28 plus IL-6/TGF-β/IL-1β (iNKT17-skewing conditions). The cells were restimulated with PMA/ionomycin, and the expression of indicated cytokines and transcription factors was examined with intracellular cytokine staining and ELISA **(A,B)**, or quantitative PCR **(C)**. Representative FACS plots and cumulative results are shown. NS, not significant, *P < 0.05; **P < 0.01; ***P < 0.001 determined by unpaired *t*-test, two-tailed (*n* = 3). **(C)** iNKT17-related mRNA profiles were measured by quantitative PCR. The expression of mRNA in each sample was normalized against the level of β-actin. The data shown are from three independent experiments, and the values from WT are arbitrarily set as 1.0. UD, undetectable.

Invariant NKT cells can be induced to produce IL-17A when activated in the presence of IL-6, TGF-β, and IL-1β (11). To determine whether c-Maf is also critical for such polarization of iNKT17 cells, we expanded WT and c-Maf KO splenic and LN iNKT cells *in vitro* with α-GalCer, then stimulated the expanded iNKT cells with anti-CD3/anti-CD28 in the presence or absence of IL-6, TGF-β, and IL-1β. The cells were re-stimulated with PMA/ionomycin prior to intracellular cytokine staining. Expectedly, IL-6, TGF-β, and IL-1β induced the polarization of iNKT17 cells with about 10% of those cells stained positive for IL-17A. By contrast, only approximately 1% of c-Maf KO iNKT17 cells produced IL-17A (Figure 4A). This difference was even more striking in LN iNKT17 cells. About 50% of LN WT iNKT17 cells were stained positive for IL-17A, whereas only 2% of c-Maf KO LN iNKT17 cells expressed IL-17A (Figure 4B). The marked impairment in the production of IL-17 by c-Maf KO iNKT17 cells was also confirmed with ELISA (Figures 4A,B).

Invariant NKT cells also express other cytokines, including IL-17F, IL-21, and IL-22, which are subject to regulation by c-Maf in T_H_17 cells. We found that the transcript levels of IL-17F and IL-21 were also notably reduced in c-Maf KO iNKT17 cells, suggesting that dependence on c-Maf is not unique to IL-17A in iNKT17 cells (Figure 4C). A recent study showed that c-Maf interacted with Sox5 and cooperatively induced T_H_17 cell differentiation *via* induction of RORγt (19). We found that the transcript level of RORγt, but not Sox5, was also markedly reduced in c-Maf KO iNKT17 cells, indicating that the mechanism of action of c-Maf in iNKT17 cells is similar to that in T_H_17 cells (Figure 4C).

### c-Maf KO iNKT17 Cells Lose the Ability to Recruit Neutrophils to the Lungs

**Figure 5:**
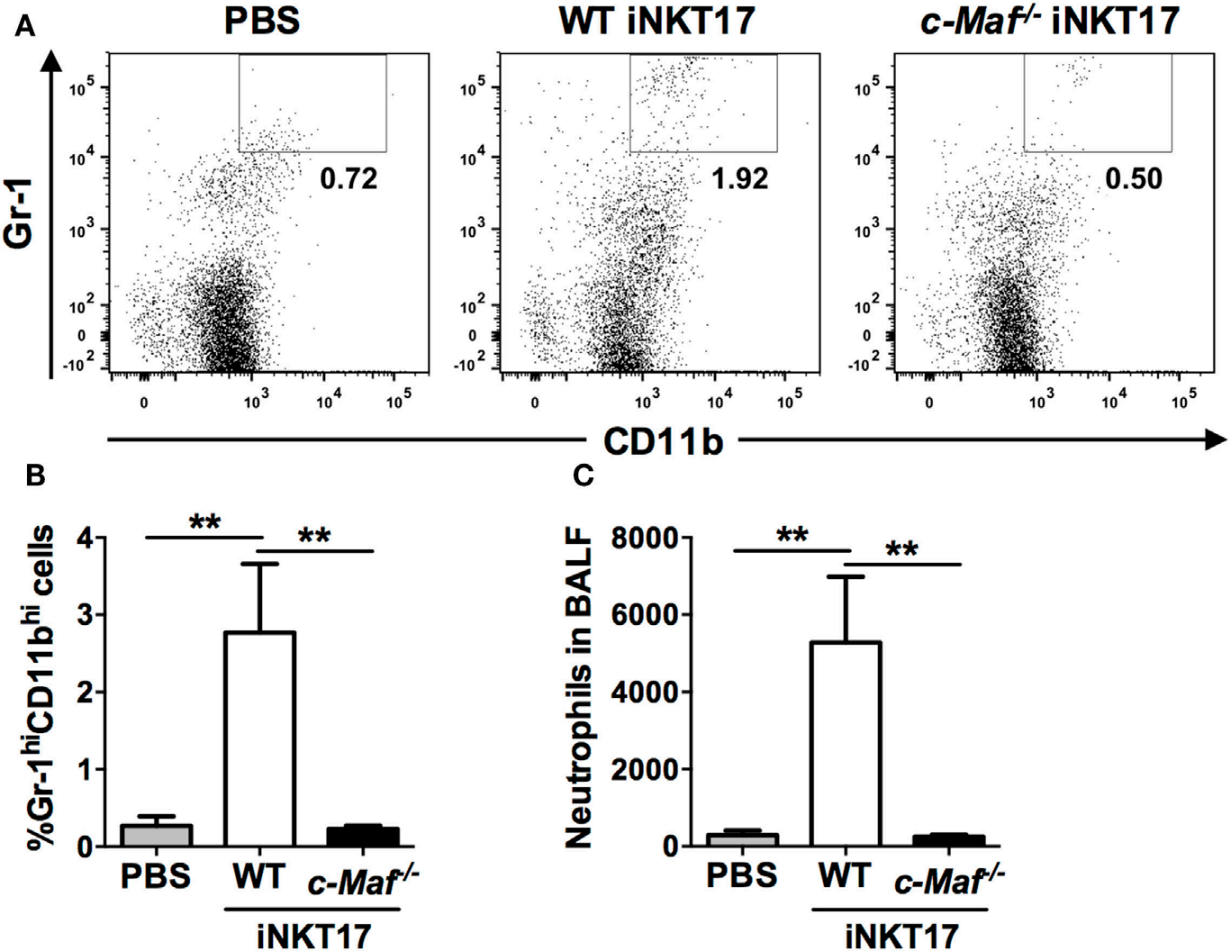
c-Maf knockout (KO) Invariant natural killer T (iNKT) 17 cells lose the ability to recruit neutrophils to the lungs. Fifty thousand wild-type (WT) or c-Maf KO iNKT17 cells, or PBS were injected into the trachea of naive C57BL/6J mice (six mice per group), which were sacrificed 2 days later. Neutrophils (Gr-1^Hi^CD11b^Hi^) in bronchoalveolar lavage were identified by FACS. Representative FACS plots are shown in **(A)**. The percentage **(B)** and absolute number **(C)** of neutrophils are also shown. **P < 0.01 determined by unpaired *t*-test, two-tailed (*n* = 6).

Invariant NKT17 cells are known to drive neutrophilic airway inflammation (11). To examine the impact of c-Maf deficiency on iNKT17 cell-mediated airway neutrophilia, we injected *in vitro* generated WT or c-Maf KO iNKT17 cells into the trachea of naive C57BL/6J mice. The mice were sacrificed 2 days later and the presence of neutrophils (Gr-1^Hi^CD11b^Hi^) in BAL was examined with flow cytometric analysis (Figure 5A). In agreement with published data, intra-tracheal injection of WT iNKT17 cells leads to a marked increase in the numbers of neutrophils in BAL (Figures 5B,C). By contrast, c-Maf KO iNKT17 cells were completely unable to cause the infiltration of neutrophils to airways.

## Discussion

Transcription factor c-Maf is critical for IL-4 production by T_H_2 cells and critical for T_H_2 commitment (12, 13). It also plays important roles in the regulation of cytokine production in T_H_17, T_FH_, Tr1 cells, and macrophages (14–17, 22). In addition, a previous study has demonstrated that c-Maf is induced by PLZF and this PLZF/c-Maf axis positively regulates IL-4 and IL-10 production by iNKT cells (20). However, whether c-Maf can regulate other cytokine gene(s) or functions in iNKT cells is not fully understood. Moreover, the roles of c-Maf in iNKT cell-mediated inflammation have not yet been addressed. Our data have established a critical role of c-Maf in promoting the expression of IL-17 by iNKT cells. This function of c-Maf is very likely mediated indirectly through the induction of RORγt. Hence, the knowledge gained from this study will bring important insight into the role of glycolipid antigen-c-Maf-IL-17A axis in iNKT cells.

The level of c-Maf transcript in unstimulated iNKT cells is comparable to that in anti-CD3-restimulated T_H_2 or T_H_17 cells and the percentages of c-Maf-expressing iNKT cells are unchanged after α-GalCer stimulation. These observations strongly suggest that the c-Maf expression is maintained at a basal level in iNKT cells, allowing them to rapidly respond to glycolipid antigens and produce a cytokine surge.

To further investigate the role of c-Maf in iNKT cells, we use CD45.1^+^ c-Maf-deficient fetal liver cells to reconstitute the hematopoietic system of WT C57B6/J mice. c-Maf plays a critical role in erythropoiesis that accompanies erythroblastic island formation in the fetal liver (21). Therefore, the number of fetal liver cells is significantly lower in *c-Maf*^−^*^/^*^−^ embryos than in WT embryos. Moreover, the frequency of c-Maf homozygous KO embryos is usually lower than WT or heterozygous embryos within the same pregnancy. It is time-consuming; at least two months per one reconstitution is needed to generate a sufficient number of c-Maf-deficient reconstituted mice to conduct the experiments. Thus, c-Maf conditional KO mice (23) could be employed in the future studies.

We found that the percentage of splenic iNKT cells was lower in WT reconstituted mice (~0.6%) compared to un-manipulated WT C57BL6/J mice (~1%). The reconstituted iNKT cells were also less robust in producing cytokines (data not shown). Nevertheless, c-Maf deficiency did not affect the development and homeostasis of iNKT cells in peripheral lymphoid organs. However, we cannot exclude the possibility that c-Maf deficiency might affect the development of iNKT cells in the central lymphoid organ. It will be of great interest to examine whether c-Maf is involved in the development of iNKT cells in the thymus once c-Maf conditional KO mice are available.

Our data are the first to show that c-Maf is a positive regulator for IL-17A production in iNKT cells. As c-Maf is also expressed in iNKT cells that produce type 1 and type 2, but not IL-17, it is essential but not sufficient to drive IL-17 expression alone. c-Maf is known to directly bind to the MARE site(s) on the promoter of target genes and positively or negatively regulate target gene expression. Ciofani et al. have clearly demonstrated that c-Maf can binds to both *Il17a* and *Il17f* promoters in T_H_17 cells (18). In our study, we found that c-Maf-deficient iNKT17 cells also expressed less *Il17a, Il17f*, and *Il21*, suggesting a global defect in iNKT17 transcriptional program. Similar to T_H_17 cells, the master transcription factor of iNKT17 cells is also RORγt (24). A recent study showed that c-Maf interacted with Sox5 and cooperatively induced T_H_17 cell differentiation *via* induction of RORγt (19). In agreement with this report, the level of RORγt was also reduced in c-Maf KO iNKT17 cells, suggesting that the mechanism of action of c-Maf in iNKT17 cells is similar to that in T_H_17 cells. It will be informative to examine whether c-Maf can bind to the promoters of *Il17a, Il17f*, and *Rorc* in iNKT17 cells by chromatin immunoprecipitation (ChIP). However, we were unable to generate sufficient numbers of c-Maf KO iNKT cells for ChIP despite several attempts.

Our results indicate that c-Maf is not required for IL-4 production in iNKT cells, a finding not consistent with the previous studies demonstrating that c-Maf is required for IL-4 production in CD4^+^ NKT or iNKT cells (13, 20). The discrepancy could be due to differences in genetic backgrounds (C57BL/6J vs. BALB/c) or approaches (hematopoietic reconstitution vs. germline KO). An alternative explanation is that there could be redundant positive regulator(s) downstream of PLZF for IL-4 expression in iNKT cells. These positive regulators could compensate the IL-4 expression in c-Maf-deficient iNKT cells. Recently, Andris et al. demonstrated that c-Maf promotes the differentiation of T_FH_ cells and showed that c-Maf-deficient T_H_2 or T_H_17 cells still can produce IL-4 or IL-17, respectively (25). These findings suggest that c-Maf plays an essential role in IL-17A expression in iNKT17 cells. It is still unclear whether c-Maf can interact with PLZF. Thus, it will be informative to examine the interaction between c-Maf and PLZF in iNKT17 cells.

Several studies have demonstrated that iNKT cells are an important source of T_H_17 type cytokines during airway inflammation. In mice, Michel et al have firstly identified an IL-17-producing NK1.1^−^ iNKT cell population that is more frequent in the lung and responsible for neutrophil recruitment after α-GalCer administration (9). Another study shows that iNKT cells are required for airway inflammation induced by environmental antigens existing in house dust extracts (HDEs). Lung iNKT cells produced not only T_H_2 cytokines but also T_H_1 cytokines and IL-17A after challenge with OVA/HDE. The IL-17A production was especially prominent in lung CD4^−^ iNKT cells. In addition, there was a synergistic activation between iNKT cells and conventional CD44^high^ memory CD4 T cells in OVA/HDE-vaccinated mice (26). It is also reported that polyinosinic-polycytidylic acid [poly(I:C)] plus α-GalCer could promote the IL-17A production by CD4^−^NK1.1^−^ iNKT cells. Their intratracheal administration resulted in increased airway hyper-reactivity, neutrophilia in BAL and airway inflammation, suggesting that airway inflammation could be further aggravated by viral induced iNKT17 cells (27). Therefore, suppressing IL-17A production by iNKT17 through targeting c-Maf can be therapeutic in airway inflammation.

## Ethics Statement

This study was carried out in strict accordance with the recommendations in the Guide for the Care and Use of Laboratory Animals of the National Institutes of Health. The protocol was approved by the Institutional Animal Care and Use Committee (IACUC) of National Taiwan University College of Medicine (permit number: 20140426).

## Author Contributions

J-SY, KY, ST, and S-CM designed the experiments; J-SY performed the primary experiments; MH and SO assisted with the experiments; J-SY analyzed the results and made the figures; J-SY and S-CM wrote the manuscript.

## Conflict of Interest Statement

The authors declare that the research was conducted in the absence of any commercial or financial relationships that could be construed as a potential conflict of interest.
